# Chimeric RNAs reveal putative neoantigen peptides for developing tumor vaccines for breast cancer

**DOI:** 10.3389/fimmu.2023.1188831

**Published:** 2023-09-06

**Authors:** Brandon Mistretta, Sakuni Rankothgedera, Micah Castillo, Mitchell Rao, Kimberly Holloway, Anjana Bhardwaj, Maha El Noafal, Constance Albarracin, Randa El-Zein, Hengameh Rezaei, Xiaoping Su, Rehan Akbani, Xiaoshan M. Shao, Brian J. Czerniecki, Rachel Karchin, Isabelle Bedrosian, Preethi H. Gunaratne

**Affiliations:** ^1^ Department of Biology & Biochemistry, University of Houston, Houston, TX, United States; ^2^ Department of Breast Surgical Oncology, University of Texas, MD Anderson Cancer Center, Houston, TX, United States; ^3^ Department of Medicine, Houston Methodist Research Institute, Houston, TX, United States; ^4^ Department of Pathology, The UT MD Anderson Cancer Center, Houston, TX, United States; ^5^ Department of Bioinformatics & Computational Biology, University of Texas, MD Anderson Cancer Center, Houston, TX, United States; ^6^ Biomedical Engineering Department, Institute for Computational Medicine, Johns Hopkins School of Medicine, Baltimore, MD, United States; ^7^ Department of Molecular & Cellular Biology, Baylor College of Medicine, Houston, TX, United States; ^8^ Human Genome Sequencing Center, Baylor College of Medicine, Houston, TX, United States; ^9^ Department of Breast Oncology, H. Lee Moffitt Cancer Center, Tampa, FL, United States

**Keywords:** RNA fusions, chimeric RNAs, neoantigens, immunopeptides, tumor peptide vaccines

## Abstract

**Introduction:**

We present here a strategy to identify immunogenic neoantigen candidates from unique amino acid sequences at the junctions of fusion proteins which can serve as targets in the development of tumor vaccines for the treatment of breastcancer.

**Method:**

We mined the sequence reads of breast tumor tissue that are usually discarded as discordant paired-end reads and discovered cancer specific fusion transcripts using tissue from cancer free controls as reference. Binding affinity predictions of novel peptide sequences crossing the fusion junction were analyzed by the MHC Class I binding predictor, MHCnuggets. CD8+ T cell responses against the 15 peptides were assessed through in vitro Enzyme Linked Immunospot (ELISpot).

**Results:**

We uncovered 20 novel fusion transcripts from 75 breast tumors of 3 subtypes: TNBC, HER2+, and HR+. Of these, the NSFP1-LRRC37A2 fusion transcript was selected for further study. The 3833 bp chimeric RNA predicted by the consensus fusion junction sequence is consistent with a read-through transcription of the 5’-gene NSFP1-Pseudo gene NSFP1 (NSFtruncation at exon 12/13) followed by trans-splicing to connect withLRRC37A2 located immediately 3’ through exon 1/2. A total of 15 different 8-mer neoantigen peptides discovered from the NSFP1 and LRRC37A2 truncations were predicted to bind to a total of 35 unique MHC class I alleles with a binding affinity of IC50<500nM.); 1 of which elicited a robust immune response.

**Conclusion:**

Our data provides a framework to identify immunogenic neoantigen candidates from fusion transcripts and suggests a potential vaccine strategy to target the immunogenic neopeptides in patients with tumors carrying the NSFP1-LRRC37A2 fusion.

## 1 Introduction

Tumor vaccines capable of promoting immune response have the potential to make significant contributions to the treatment and prevention of cancer. The antigenic repertoire that arises during tumorigenesis through somatic alterations in tumors provides a plethora of non-self-antigens (neoantigens) that can form the basis of vaccination-based cancer immunotherapies. Many of the neoantigens discovered have been shown to be capable of inducing anti-tumor immune responses with minimal side effects in the treatment setting (1, 2). Neoantigen load has been reported to be strongly correlated with clinical response to immunotherapy (3) and high somatic mutational burden. A high density of candidate neoantigens have also been shown to improve survival in patients treated with immune checkpoint blockades in non-small cell lung cancer (NSCLC) (4) and melanoma (5, 6). However, many neoantigens caused by non-synonymous mutations are patient specific, thus can only be used as personalized vaccines and not available as an ‘off the shelf’ option for treatment that would facilitate widespread adoption (7). Therefore, identification of shared neoantigens generated through aberrant transcripts which are prevalent in cancer patients would help overcome one of the current challenges in the advancement of vaccination-based cancer immunotherapies.

Much of the work on neoantigens relates to single nucleotide variants (SNV) and small insertions and deletions (indel) (8). However, for cancers with a low to moderate mutation burden, such as breast cancer, these approaches provide a limited neoantigen repertoire that can be harnessed for therapeutic cancer vaccines. Non-mutated, over-expressed peptides have thus been of interest in this context, with much of the clinical research focused on peptides derived from HER2-Neu (9, 10). Additional approaches that expand the available immunogenic peptides for use in cancer vaccines in these tumors with a limited repertoire of neoantigens derived from non-synonymous mutations is needed if this promising immunotherapy strategy is to be fully utilized clinically.

Here, we focused on identifying neoantigens in fusion transcripts from two separate genes identified from RNA-sequencing (RNA-seq) data of breast cancer samples. The unique sequences at the fusion junctions form new open reading frames (ORFs) that can result in fusion proteins representing a hybrid of the two founding genes and/or truncated versions of the two wild type proteins due to premature termination of the 5’-gene yielding a unique amino acid sequence in the C-terminus and novel N-terminal region in the 3’gene. Our main objective was to discover whether such intergenic spliced chimeric mRNA can provide novel neoantigens that can be processed and presented by the major histocompatibility complex (MHC) Class I peptides to target CD8^+^ T cells. The ultimate goal of this work is to establish a framework for using immunogenic neopeptides generated from the novel amino acids at the fusion junctions of chimeric RNAs for the development of “off the shelf” tumor vaccines for breast cancer.

## 2 Materials and methods

### 2.1 Samples and controls

All tissue samples were obtained from archival formalin fixed, paraffin embedded (FFPE) blocks under a protocol approved by the MD Anderson Cancer Center Institutional Review Board. Tumor samples were obtained from women who met the following criteria: i) newly diagnosed breast cancer, ii) no prior history of breast cancer (primary disease), iii) undergoing surgery as the initial treatment modality, iv) no prior receipt of chemotherapy. In addition, only tumors from women with no known germline mutations and without a significant family history were included in order to enrich for sporadic cancers. Stage was not specifically selected for, however all patients had non-metastatic disease. Seventy-five cases from cancer patients were used, 25 from each of the 3 main clinical subtypes: i) estrogen and/or progesterone positive and HER2 negative (referred to as hormone receptor [HR] positive), ii) HER2 positive regardless of HR status and iii) HR negative and HER2 negative (TNBC; triple negative). Four breast tissue samples from women without a cancer diagnosis were used as controls.

### 2.2 RNA extraction

RNA extraction was conducted using the Ambion Recoverall Total Nucleic Acid isolation kit (cat# AM19750, ThermoFisher) following the manufacturer’s recommendations. Briefly, tissue cores were crushed, placed in 1.5ml tubes and washed three times with 100% xylene for 10 min. Tissues were then washed in 100% ethanol twice for 10 min followed by one wash in 95% ethanol for 10 min and another wash in 10% PBS, then allowed to air dry for 5 min. Tissues were then incubated in protease digestion buffer at 50°C for 3 hours followed by a 15 min incubation at 80°C after which tissues were stored in -20°C until RNA isolation. At the time of RNA extraction, isolation additive and ethanol mix were added to each sample and placed into the filter cartridge followed by centrifugation for 30 sec at 10,000xg. This was repeated 3 times followed by the addition of wash solutions and centrifugation. DNase was then added to the filter cartridge and incubated at room temperature for 30 min. RNA was then eluted by adding nuclease free water to the center of the filter cartilage, incubating for 5 min and centrifugation at maximum speed for 1 min. RNA was then stored at -80°C.

### 2.3 FFPE RNA quality control

Extracted RNA samples underwent quality control assessment using the RNA tape on a Tapestation 4200 (Agilent, RRID : SCR_019398). DV200 was calculated as the percentage of RNA fragments that are >200 nucleotides in size. All samples had a DV200 >30% which is the recommended cutoff for RNA sequencing (Illumina Technical Pub. No. 470-2014-001,2016). Samples were then quantified with Qubit Fluorometer (ThermoFisher) for input into library preparation.

### 2.4 Transcriptome sequencing

The RNA libraries were prepared and sequenced at the University of Houston Seq-N-Edit Core per standard protocols. RNA libraries were prepared with the TruSeq RNA Exome kit (Illumina) using 30 ng input RNA. RNA was fragmented, reverse transcribed into cDNA and ligated with sequence adaptors. The size selection for libraries was performed using SPRIselect beads (Beckman Coulter). Enrichment for coding RNA was performed by coding region specific biotinylated capture probes and selected by streptavidin magnetic beads. Library purity was analyzed using the DNA 1000 tape on a Tapestation 4200 (Agilent, RRID : SCR_019398) and quantified with Qubit Fluorometer 2.0 (ThermoFisher, RRID : SCR_020553). The prepared libraries were pooled and sequenced using the NextSeq 500 (Illumina, RRID : SCR_016381); generating ~15 million 2×76 bp paired end reads per sample.

### 2.5 RNA fusion detection

The RNA-seq raw fastq data was processed with CLC Genomics Workbench 20 (Qiagen). The Illumina sequencing adaptors were trimmed, and reads were mapped to the human reference genome hg38 Refseq GRCh38.p9 from the Biomedical Genomics Analysis Plugin 20.0.1 (Qiagen). Read alignment was represented as integer counts by using parameters of mismatch cost 2, insertion cost 3, deletion cost 3, length fraction 0.8, similarity fraction 0.8, max of 10 hits for a read. Integer read counts were normalized by Trimmed Means of M-values (TMM) algorithm (11). RNA fusions were detected using the detect fusion gene algorithm under the parameters of minimum length of unaligned sequence 15, maximum distance to exon boundary 10, maximum distances for broken pair fusions 1,000, assumed error rate 0.001, promiscuity threshold 7. The algorithm identifies fusion events based on the number of fusion crossing reads and fusion spanning reads. Refine fusion gene tool was used to re-count the number of fusion crossing reads and the novel RNA seq reads mapped against the fusion reference created in detect fusion genes. The fusion list was further refined by excluding those that were detected in both normal breast tissue controls and in paired adjacent normal tissue samples. Details of the false positive and negative filters applied are shown below.

**False Positive filter:** To reduce the false positive rates of ~50% associated with the majority of fusion callers that rely only on discordant paired end reads we introduced a filter that first extracts fusion candidates based on discordant paired end reads and then filter out fusion candidates that are not supported by at least 1 junction crossing read that has to be split to map on two different genes on the reference genome.

**False Negative filter:** To capture fusions associated with small sub populations of cells in pre-cancerous lesions and/or ‘cancer stem cells’ driving drug resistance and disease recurrence we relaxed filters that eliminate candidates based on read numbers and included fusions supported by junction crossing split reads mapping on two different genes supported by at least 1 read in three independent patients across the 3 subtypes studies. Additionally, using the CLC Genomics Workbench, we included a secondary alignment of unmapped RNA-seq reads to a fusion reference sequence created in the initial detect fusion genes pipeline. This decreased the number of false negatives discovered in other fusion callers.

### 2.6 Validation of junction sequence

cDNA from whole transcriptome sequencing underwent PCR amplification across the *NSFP1-LRRC37A2* fusion junction site using Forward Primer (5’-GCCTGCAAGTGACGAGAG-3) and Reverse Primer (5’-CGGTCCAACTGTATGCTTTC-3’). DreamTaq DNA polymerase (ThermoFisher Scientific; Cat.# EP0701) was used in a 30-cycle PCR reaction. Amplicon size was analyzed using the High Sensitivity DNA 1000 tape on a Tapestation 4200 (Agilent, RRID : SCR_019398).

### 2.7 Validation of junction sequence: cloning & sanger sequencing

The PCR amplicon was inserted in to a pJET1.2 vector as per the sticky-end cloning protocol provided by the manufacturer (CloneJET PCR Cloning Kit; ThermoFisher Scientific; Cat.# K1232). The ligation mixture was directly transformed to provided competent cells and plated on Ampicillin-LB agar plates. Plates were incubated overnight at 37°C. After incubation, 4 colonies were selected per plate to confirm the DNA insert. A PCR was performed to validate the junction sequence using the primers for *NSFP1-LRRC37A2*. Colonies expressing the amplicon were grown in Ampicillin LB broth at 37°C in a shaking incubator overnight. Plasmids extraction from the bacterial cultures was carried out using manufacturer supplied protocols (QIAprep Spin Miniprep Kit; Qiagen; Cat.# 27104) and were verified using Sanger sequencing.

### 2.8 Neoantigen predictions

Our neoantigen prediction pipeline is described in Shao et al. (12). Neopeptide regions were delineated from the 2 major ORFs predicted from the *NSFP1* [Exon 1-13] - *LRRC37A2* [Exon 2-14] fusion. To assess the immunogenicity of our predicted neopeptides in relation to 118 MHC class I haplotypes found in humans, we utilized a neoantigen prediction platform, MHCNuggets. Peptides of 8 amino acids encompassing two major ORFs generated from the *NSFP1-LRRC37A2* fusion were analyzed. The HLA genotypes extracted from RNASeq fusion caller from the 75 samples served as input to MHCnuggets to predict the MHC class I binding potential (IC50 nM) of each peptide region from wild-type and neoantigen peptide regions of two truncated proteins. Neoantigen candidates meeting an IC50 affinity < 500 nM were subsequently ranked based on MHC binding. Anchor and auxiliary anchor residues for neopeptide-HLA class I allele pairs were evaluated by the SYFPEITHI online tool (13).

### 2.9 Peptide library generation

The peptide library consisted of 15 neoantigenic 8-mer peptides discovered from the *NSFP1*- Exon 1-13 truncation ORF and *LRRC37A2*-Exon 2-14 truncation ORF and was synthesized and purified using standard solid-phase synthetic peptide chemistry and Reverse Phase High Performance Liquid Chromatography (ThermoFisher Scientific PEPotec). These peptides were reconstituted to 1 mg/mL concentrations under sterile conditions. An 8-mer peptide used by the manufacturer to standardize the peptide library which was confirmed to be a peptide of no biological significance was used as a Negative Peptide Control (NCP) to validate the effect of stimulation by a synthetic peptide. A commercially available Cytomegalovirus (CMV) peptide pool (MabTech; Cat.# 3619-1) containing 42 peptides from the Cytomegalovirus where 28 of the peptides are MHC class I restricted and 14 are MHC class II restricted was used as the positive control.

### 2.10 Human primary cells

The HLA class C07:02 matched human Peripheral Blood Mononuclear Cells (PBMCs) from a healthy donor were acquired (STEMCELL Technologies) and were stored in liquid nitrogen until use.

### 2.11 Culture medium

Complete media consisted of RPMI-1640 growth media with L-glutamine (Gibco; Cat.# 61870036) supplemented with 10% heat-inactivated fetal bovine serum (GenDEPOT; Cat.# F0601-050), 0.1 mmol/L nonessential amino acids (Corning; Cat.# 25-025-CI), 10ug/ml Cellmaxin (GenDEPOT; Cat.# C3319-006), and 0.5 mg/mL Amphotericin B (Gibco; Cat.# 15290026).

### 2.12 *In vitro* stimulation of PBMCs using peptides

PBMCs were retrieved from liquid nitrogen, thawed in a water bath at 37°C, and washed with culture medium warmed to 37°C, as previously described in the primary cell thawing protocol by Stem Cell Technologies. Cells were incubated at 37°C, 5% CO_2_ for 24 hours (Cell Resting). After resting, cells were seeded at a concentration of 1 × 10^6^/mL in 6-well plates with culture medium containing IL-2 (10 IU/ml), IL-7 (10 ng/ml), and IL-15 (10 ng/ml). The cells of the Negative (Unstimulated) control (NC) wells not treated with any peptides but were supplemented with the growth medium and cytokines required for growth and proliferation and were maintained at the same growth conditions as the cells of wells treated with the neoantigenic peptides. The cells of the CMV positive control wells were treated with 1μg/ml of the CMV peptide pool and were supplemented with media and growth conditions identical to that of the test peptide wells. The 15 neaoantigenic 8-mer test peptides were added to the respective wells at 2 μg/ml and the plates were incubated at 37°C, 5% CO_2_ for 4 days On day 5, 50% of the medium was replaced with fresh medium, and cells were cultured for an additional 5 days. A second round of peptide restimulation was carried out with the corresponding peptides coupled with the cytokine medium before the cells were used for the ELISpot assay.

### 2.13 Isolation of CD8+ T cells from PBMCs

On Day 13, untouched CD8^+^ T cells were isolated from PBMCs by magnetic negative selection using the MojoSort™ Human CD8^+^ T Cell Isolation Kit (BioLegend; Cat.# 480012) following the manufacturer’s instructions.

### 2.14 IFN-γ ELISpot assay

To evaluate peptide stimulated CD8^+^ T cell immune response, IFN-γ production by cells stimulated with the predicted neoantigenic peptides was quantified using a commercially available Human IFN-γ- ELISpot kit (CTL ImmunoSpot, Cellular Technology Ltd), following the instructions of the manufacturer. The plate was read with an ELISpot reader (CTL counter, Cellular Technology Ltd). The cell culture medium used to incubate the cells in the ELISpot plate was augmented with anti-CD28 antibody (1μg/ml) and corresponding peptides (2μg/ml).

### 2.15 Statistical analysis

Positive response to the assay was defined using a threshold minimum of 20 Spot Forming Colony Units (SFC)/10^6^ cells in experimental wells after subtracting the unstimulated background (Mean number of SFUs generated by the NC wells). To compare immune responses generated by the neonatigenic peptides, SFUs generated by the wells stimulated with the neoantigenic peptides were compared with that of the wells stimulated with CMV peptide pool. ELISpot data were analyzed by Mann-Whitney U Test, without correction for multiple comparisons, using GraphPad Prism 9.0 (RRID : SCR_002798). Each row was analyzed individually, without assuming consistent standard deviation. Data are represented as mean ± SEM. For all analyses, significance threshold was considered as *, P ≤ 0.05.

## 3 Results

### 3.1 Twenty highly prevalent fusion transcripts were discovered across 3 breast cancer subtypes

With the goal of discovering RNA-fusions that can be targeted for neoantigen peptide candidates, we performed RNA-Sequencing of triple negative (TNBC), HER2+ and hormone receptor positive (HR+) breast cancer samples (n=25 each). Mining the sequence reads (i) that were discarded due to discordant paired-end reads and (ii) that were supported by split-reads (junction crossing reads) we found a large number of chimeric fusion RNAs. These were then cross referenced with the TCGA Multi-Center Breast Cancer Dataset. We uncovered 20 fusion RNAs with high prevalence across the set of 75 tumor samples and also detected in 1 or more of the TCGA samples. To eliminate false positives, we also required a given fusion to be present within more than one dataset discovered by an independent fusion caller (CLC Genomics Workbench and University of Chicago fusion caller). **Table 1** shows the comprehensive list of fusion transcripts with the number of samples in each subtype that was found to carry the fusion in the tumor.

**Table 1:** Top 20 novel prevalent chimeric RNAs discovered in TNBC, HER2+, and HR+ patient sample gene fusions after comparison to normal samples.

RNA FUSIONS (BREAST CANCER)	EXON Boundaries	TNBC Fusions		HER2+ Fusions		HR+ Fusions		TCGA (Breast Tumors)
		# of Fusion Positive Samples	Avg. # Junction Crossing Reads	# of Fusion Positive Samples	Avg. # Junction Crossing Reads	# of Fusion Positive Samples	Avg. # Junction Crossing Reads	# Samples
*NSFP1-LRRC37A2*	Exon 1-13| Exon 2-14	2	218	2	274	5	217	5
*F8-CLIC2*	Exon 1|Exon 2-6	1	50	4	6	3	2	1
*KIAA0753-PITPNM3*	Exon 1-16 | Exon 2-20	0	25	3	2	4	3	1
*PRKCH-FLJ22447*	Exon 1-12 | Exon 2-3	3	24	5	1	3	1	26
*PACSIN2-ARFGAP3*	Exon 1-11 | Exon 2-6	2	15	2	1	3	1	1
*UBE3C-DNAJB6*	Exon 1 | Exon 2-8	1	13	0	0	1	2	2
*NCOR2-UBC*	Exon 1-15 | Exon 1	0	13	1	1	2	2	3
*GALK2-FGF7*	Exon 1-10 | Exon 3-4	1	11	1	4	2	3	1
*ARIH2-SLC25A20*	Exon 1-5 | Exon 5-9	0	8	0	0	1	2	2
*B4GALT1-SMU1*	Exon 1-2,3| Exon 2-12	2	7	1	2	1	2	1
*WNK1-ERC1*	Exon 1-24|Exon 6-18	1	7	0	0	0	0	1
*SCCPDH-CNST*	Exon 1-5| Exon 4-9	1	6	0	0	0	0	1
*NOXRED1-TMED8*	Exon 1-5 | Exon 2-6	1	3	1	1	0	0	1
*ACAP2-XXYLT1*	Exon 1-21 | Exon 3-4	0	3	1	1	0	0	1
*MBD5-ORC4*	Exon 1-2|Exon 2-14	1	2	1	2	0	0	2
*UBE2G1-ANKFY1*	Exon 1-3 | Exon 3-25	1	2	6	0	1	1	1
*AKT3-SDCCAG8*	Exon 1|Exon 7-18	0	1	0	0	1	1	3
*BACE2-FAM3B*	Exon 1-8| Exon 2-7	0	1	0	0	1	1	3
*ADCY9-SRL*	Exon 1-2 | Exon 2-6	0	1	1	2	0	0	6
*TMCO3-TFDP1*	Exon 1-7 | Exon 3-12	1	7	0	0	0	0	6

To remove false positive discoveries the fusions was required to be found in an independent dataset (TCGA Breast Cancer dataset). Exon boundaries from the fusion junction site between the 2 genes, the number of tumor samples positive for each fusion (n=25) for each subtype, and the average number of junction crossing reads identified from the positive sample are shown.

The average number of junction crossing reads as well as the exon boundaries of the 5’ and 3’ genes in both our dataset and TCGA are also presented. Of the 20 novel fusions found, 4 were identified with a frequency of 10% or greater in the MD Anderson Cancer Center (MDACC) cohort. The *NSFP1*- *LRRC37A2* fusion transcript was selected for further study based on the fact that it was associated with the highest number of junction crossing reads (TNBC=218, HER2+=274, HR+=217), and detected with highest frequency across the 75 tumor samples (9/75 = 12%), (TNBC=2 samples, Her2+=2 samples and HR+=5 samples). Furthermore, it was also present in 5 samples in the TCGA breast cancer dataset previously analyzed with filters that traditionally exclude fusions found in adjacent normal tissue. TCGA, however, did not remove fusions from cancer free controls similar to what was done in this study.

### 3.2 Exon boundaries of *NSFP1-LRRC37A2* Fusion Maps to Exon 13 of *NSFP1* (5’-boundary) and Exon 2 of *LRRC37A2* (3’-boundary)

**Figure 1:**
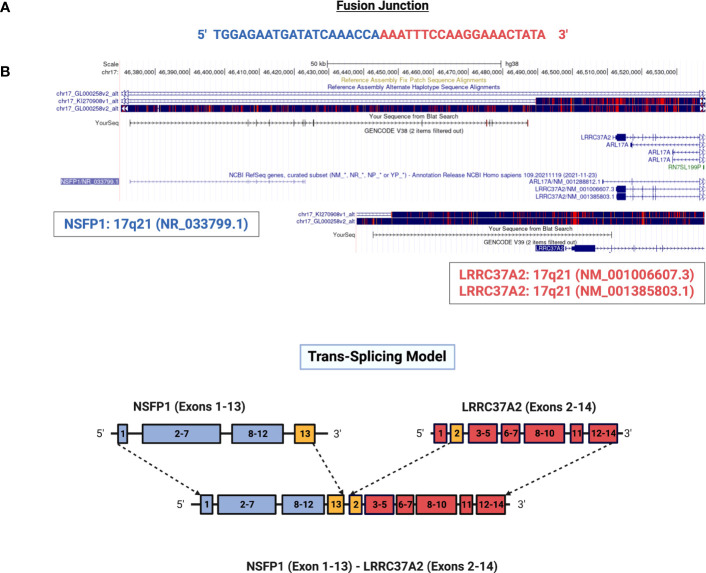
Genomic mapping of junction crossing reads for *NSFP1-LRRC37A2.* **(A)** The fusion junction sequence. The sequence of the junction-crossing read extracted from 986 sequence reads from 75 samples (25 Tumor samples – 3 subtypes) is shown. The segment of the reads that map to *NSFP1* and *LRRC37A2* is shown in Blue and Red respectively. **(B)** A model of the novel fusion transcript *NSFP1-LRRC37A2*. The junction site is shown in green between exon 13 of *NSFP1* and exon 2 of *LRRC37A2*.

**Figure 2:**
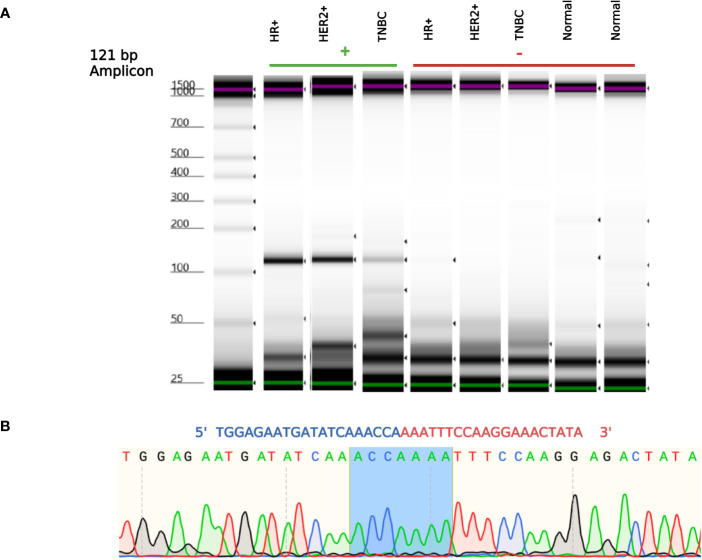
*NSFP1-LRRC37A2* Fusion PCR validation. **(A)** One fusion junction positive sample from each subtype, was chosen to be validated by PCR. Capillary gel electrophoresis was used to detect the 121 bp amplicon fragment, representing the *NSFP1-LRRC37A2* fusion. **(B)** The sanger sequencing chromatogram of the PCR amplicons cloned into plasmids and sequenced. The junction site of the fusion between exon 13 of *NSFP1* and exon 2 of *LRRC37A2* is shown in blue in the chromatogram.

*NSFP1* (N-ethylmaleimide sensitive factor, vesicle fusing ATPase, transcript variant 1 pseudogene) and LLRC37A2 (Leucine Rich Repeat Containing 37 Member A2) are located in 17q21.31. To compile the *NSFP1-LRRC37A2* fusion junction, we mapped the consensus junction sequence compiled from the complete set of junction crossing reads extracted from fusion positive samples to hg38 Refseq GRCh38.p9. The 5’-boundary of *NSFP1-LRRC37A2* was found to be located on Exon 13 of *NSFP1* (NR_033799.1) and the 3’ – boundary mapped to Exon 2 (NM_001006607.3) of *LRRC37A2* located immediately 3’ to NSP1 on the coding strand of both genes. The boundaries were consistent and supported by 986 junction-crossing reads (TNBC=218, HER2+=274 and HR+=217) with the breakpoint sequence always **AAACCA-3’** on the *NSFP1* gene and **5’-AAATTC** on LRRC37A2. The 5 samples found to be positive for *NSFP1-LRRC37A2* fusion in the TCGA dataset (an independent set of samples) also contained the same exon boundaries. The fusion junction and the exon boundaries model for the *NSFP1-LRRC37A2* fusion are shown in **Figure 1**. The consensus junction sequence and the cDNA for the fusion transcript are shown in **Supplemental Figure 1**. The fusion junction supported by 986 junction crossing reads was validated by amplicon PCR assay as shown in **Figure 2**. We expected a 121bp PCR fragment from the PCR amplicon generated using a Forward Primer located on *NSFP1* (5’-GCCTGCAAGTGACGAGAG-3) and Reverse Primer located on *LRRC37A2* (5’-CGGTCCAACTGTATGCTTTC-3’). The PCR amplicons of 121bp cloned to the positive selection cloning vector were Sanger sequenced to further validate the presence of the fusion junction. The chromatogram acquired through Sanger sequencing is also shown in **Figure 2**. The same exon boundary of *NSFP1* Exon 13 *LRRC37A2* Exon 2 identified by the CLC Genomics workbench 20.0 (Qiagen) on the breast cancer dataset presented here was also found in the fusions uncovered TCGA and MDACC datasets.

### 3.3 Novel fusion junctions from the *NSFP1-LRRC37A2* fusion transcript variants contain two major ORFs generating two truncated proteins

**Figure 3:**
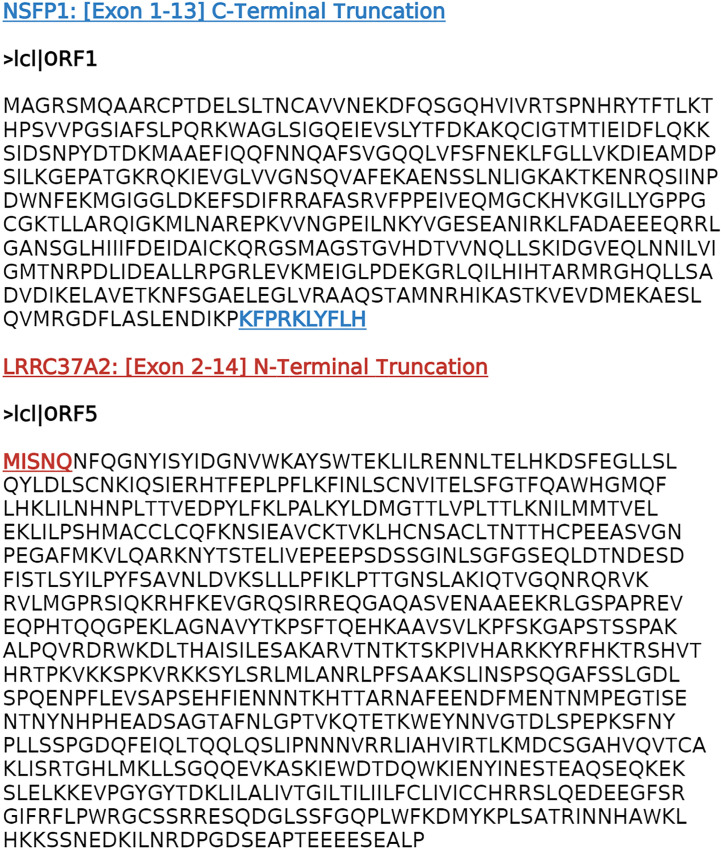
*NSFP1- LRRC37A2* fusion transcript predicted ORFs. The cDNA sequence generated from the *NSFP1-LRRC37A2* fusion model was analyzed through the NCBI-Open Reading Frame (ORF) Finder. Two major ORFs consistent with two truncated proteins that are predicted from the *NSFP1* [Exon 1-13] - *LRRC37A2* [Exon 2-14] fusion transcript were uncovered. The *NSFP1* [Exon 1-13] 3’-end truncation yielded an ORF of 500 amino acids. The *LRRC37A2* [Exon 2-14] 5’-end truncation yielded an ORF of 835 amino acids.

**Figure 4:**
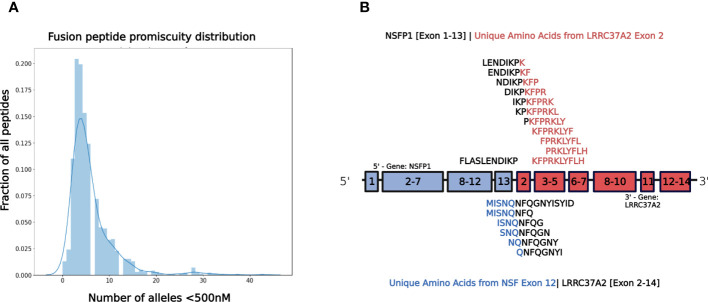
*NSFP1-LRRC37A2* Fusion Model and Immunogenic Neoantigen Peptide Fragments. **(A)** The distribution model shows the promiscuity of peptides binding to MHC Class 1 alleles. The X-axis is the number of MHC Class 1 alleles and the Y-axis is the number of total peptides found. While a majority of peptides bind less than 10 MHC Class 1 alleles, a small fraction binds to >20, which are considered to be highly promiscuous. **(B)** The unique peptide junction regions predicted from the *NSFP1* [Exon 1-13] -*LRRC37A2* [Exon 2-14] fusion transcript are shown here. The immunogenic peptides generated through MHC Class I binding predictor (MHCnuggets) from the *NSFP1* [Exon 1-13]-C-Terminal truncation are shown above the fusion transcript model and the *LRRC37A2* [Exon 2-14]-N-Terminal truncation are shown below. Amino acid residues from *NSFP1* and *LRRC37A2* are shown in (blue) and (red) respectively. The unique amino acids formed at the fusion junction are shown in (black).

The major open reading frames (ORFs) predicted from the *NSFP1* [Exon 1-13] *-LRRC37A2* [Exon 2-14] fusion are shown in **Figure 3**. Two regions of unique amino acid residues carrying neopeptides were uncovered from the 2 major ORFs predicted from the *NSFP1* [Exon 1-13] - *LRRC37A2* [Exon 2-14] fusion. The truncated *NSFP1* protein yielded the unique peptide fragment KFPRKLYFLH at the C-terminal end of *NSFP1* Exon 13 fused with the beginning of *LRRC37A2* Exon 2. The truncated *LRRC37A2* protein yielded the unique peptide fragment MISNQN at the N-terminal end of *LRRC37A2* Exons 2-14 (unique amino acids contributed by Exon 13 of *NSFP1*). To assess the immunogenicity of our predicted neoantigens a total 15 peptides of 8–11 amino acids extracted from the 2 major ORFs generated from the *NSFP1-LRRC37A2* fusion were processed through the neoantigen prediction platform, MHCnuggets, which evaluates binding of somatic peptides to MHC class I, antigen processing, self-similarity and gene expression (12). A total of 106 HLA genotypes served as input to MHCnuggets to predict the MHC class I binding potential (IC50nM) of each peptide region. Neoantigen candidates meeting an IC50 affinity < 500nM were subsequently ranked based on MHC binding. Anchor and auxiliary anchor residues for neopeptide-HLA class I allele pairs were evaluated by the SYFPEITHI online tool (13). These peptides were then rank ordered for binding affinity to the greatest number of MHC class I alleles (promiscuity), antigen processing, and self-similarity. To identify the most promiscuous peptides, which have been shown to be strong vaccine candidates (14), we ranked the peptides by number of HLA Class I alleles that each peptide bound to at a binding affinity threshold of IC50 <500nM. The promiscuity distribution plot for the complete set of peptides generated from the *NSFP1-LRRC37A2* fusion is shown in **Figure 4**. While many of the peptides bind to less than 10 MHC class 1 alleles, a small fraction does bind to >20 MHC alleles which were further investigated. We uncovered 10 and 5 immunogenic neoantigen peptides from the truncated NFS protein variant and the truncated *LRRC37A2* protein variant respectively. **Table 2** presents data from the selected neoepitopic regions with HLA class I IC50 affinities of < 1000nM, < 500nM and < 50nM. Previous studies have reported that predicted antigens with IC50<50 nM bind too strongly and do not initiate an immune response, so we chose to pursue MHC class I alleles with a binding affinity of IC50<500nM (15). A total of 10 different 8-mer neoantigen peptides discovered from the *NSFP1*-Exon 1-13 truncation ORF were predicted to bind to a total of 28 unique MHC class I alleles with a binding affinity of IC50<500nM (**Table 3**). A total of 5 different 8-mer neoantigen peptides discovered from the *LRRC37A2*-Exon 2-14 truncation ORF were predicted to bind to a total of 7 unique MHC class I alleles with a binding affinity of IC50<500nM. The unique set of MHC Class I alleles binding the immunogenic neoantigens from *NSFP1* and *LRRC37A2* truncations are shown in **Supplementary Table 1**.

**Table 2:** Predicted immunogenic neo-antigen peptide fragments from the *NSFP1* [Exon 1-13]-*LRRC37A2* [Exon 2-14] Fusion with MHC Class I partners.

Unique Peptide Region *NSFP1* [Exon 1-13] C-Terminal Truncation			
FLASLENDIKPKFPRKLYFLH			
*NSFP1* Exon 1-13 | Unique from *LRRC37A2* Exon 2	# of alleles<1000nM	# of alleles<500nM	# of alleles<50nM
**FPRKLYFL**	18	15	6
**KFPRKLYF**	15	13	5
NDIKP**KFP**	6	6	1
KP**KFPRKL**	5	5	2
ENDIKP**KF**	6	5	1
DIKP**KFPR**	6	5	1
IKP**KFPRK**	5	4	0
**PRKLYFLH**	5	3	3
LENDIKP**K**	3	2	0
**PKFPRKLY**	2	1	0
Unique Peptide Region *LRRC37A2* [Exon 2-14] N-Terminal Truncation			
**MISNQ**NFQGNYISYID			
Unique from *NSFP1* Exon 13 | *LRRC37A2* Exon 2-14	# of alleles<1000nM	# of alleles<500nM	# of alleles<50nM
**MISNQ**NFQ	4	4	2
**Q**NFQGNYI	4	3	2
**NQ**NFQGNY	6	5	1
**ISNQ**NFQG	4	4	1
**SNQ**NFQGN	3	2	0

Peptide fragments predicted to bind multiple MHC Class 1 alleles at IC50<1000nm, IC50<500nm, and IC50<50nm. Unique amino acids derived from the NSFP1-LRRC37A2 fusion are represented in red and blue respectively.

**Table 3:** Immunogenic neo-antigen peptide fragments from the *NSFP1* [Exon 1-13]-*LRRC37A2* [Exon 2-14] Fusion predicted to bind with MHC Class I alleles at IC50<500nM.

Unique Peptide Region *NSFP1* [Exon 1-13] C-Terminal Truncation				Unique Peptide Region *LRRC37A2*[Exon 2-14] N-Terminal Truncation	
FLASLENDIKPKFPRKLYFLH				MISNQNFQGNYISYID	
FPRKLYFL	IC50	PKFPRKLY	IC50	MISNQNFQ	IC50
HLA-B*42:01	4	HLA-C*07:02	75	HLA-A*68:23	8
HLA-B*08:01	10	**KPKFPRKL**	**IC50**	HLA-A*32:07	32
HLA-A*32:07	16	HLA-B*42:01	44	HLA-A*32:15	168
HLA-A*68:23	20	HLA-C*07:02	50	HLA-C*03:03	492
HLA-B*44:01	24	HLA-B*07:02	125	**ISNQNFQG**	**IC50**
HLA-B*07:02	43	HLA-B*07:01	326	HLA-A*68:23	30
HLA-C*14:02	51	HLA-A*32:07	477	HLA-A*32:07	59
HLA-B*53:01	62	**DIKPKFPR**	**IC50**	HLA-C*12:03	132
HLA-C*08:02	70	HLA-A*33:01	7	HLA-A*32:15	209
HLA-B*07:01	93	HLA-C*07:02	100	**SNQNFQGN**	**IC50**
HLA-B*15:02	146	HLA-A*68:23	127	HLA-A*68:23	132
HLA-C*07:02	152	HLA-A*68:01	210	HLA-A*32:07	156
HLA-A*32:15	169	HLA-A*32:07	362	**NQNFQGNY**	**IC50**
HLA-C*03:04	260	**PRKLYFLH**	**IC50**	HLA-A*30:02	13
HLA-C*03:03	279	HLA-A*68:23	25	HLA-A*68:23	94
**KFPRKLYF**	**IC50**	HLA-C*14:02	35	HLA-B*15:01	139
HLA-A*24:03	2	HLA-A*32:07	48	HLA-A*32:07	141
HLA-A*68:23	10	**NDIKPKFP**	**IC50**	HLA-A*32:15	335
HLA-A*32:07	15	HLA-B*44:01	40	**QNFQGNYI**	**IC50**
HLA-C*14:02	16	HLA-A*68:23	84	HLA-A*68:23	23
HLA-C*03:03	41	HLA-A*32:07	115	HLA-A*32:07	36
HLA-B*15:02	53	HLA-C*08:02	262	HLA-A*32:15	160
HLA-C*07:02	56	HLA-C*07:02	271		
HLA-A*32:15	71	HLA-A*32:15	332		
HLA-B*15:03	161	**ENDIKPKF**			
HLA-A*23:01	203	HLA-C*07:02	39		
HLA-B*44:01	279	HLA-B*44:01	75		
HLA-A*24:01	407	HLA-C*08:02	81		
HLA-B*27:02	410	HLA-A*32:07	169		
**LENDIKPK**		HLA-A*68:23	203		
HLA-A*68:23	250				
HLA-C*07:02	316				
**IKPKFPRK**					
HLA-A*68:23	69				
HLA-C*07:02	124				
HLA-A*32:07	159				
HLA-A*30:01	260				

Wild-type amino acids are colored (black), amino acid residues from the NSFP1-Truncation are colored (red) and residues from the LRRC37A2-truncation are colored (blue) respectively.

### 3.4 CD8+ T cell immune responses were elicited by 1 out of 15 candidate fusion neopeptides

**Figure 5:**
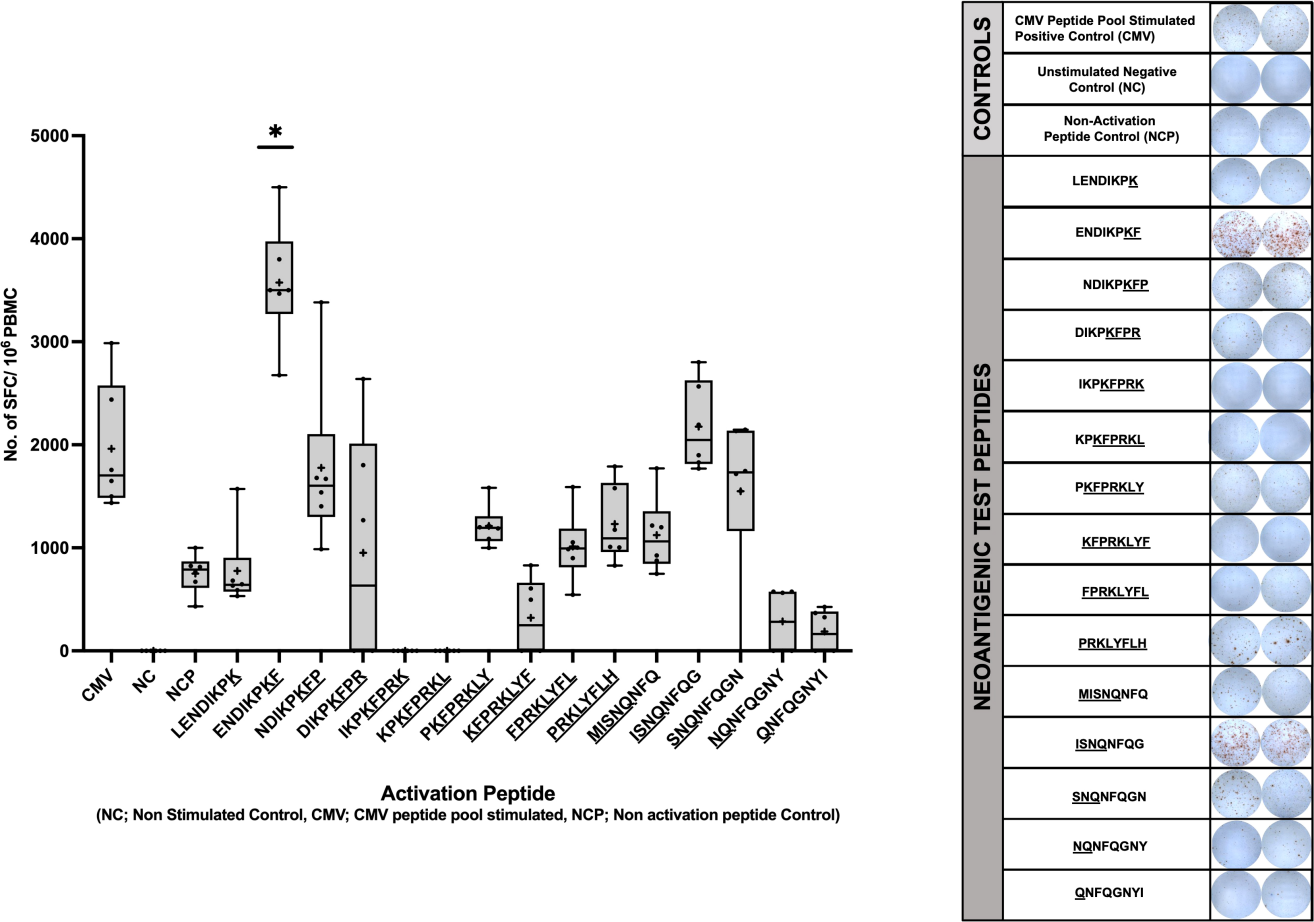
Human IFN-g ELISpot Assay using predicted immunogenic peptides of NSFP1-LRRC37A2. PBMCs from an HLA matched healthy donor were stimulated with the 15 predicted immunogenic peptides and analyzed via IFN-g ELISpot. Data represented as mean ± SEM. For the analysis, significance threshold was considered as *, P ≤ 0.05.

To determine if the predicted neopeptides induced CD8+ T cell immune responses *in vitro*, IFN-γ secretion of PBMCs was evaluated through ELISpot. The IFN-γ secretion of the cells stimulated with the 15 neopeptides were compared to that of PBMCs stimulated with a CMV peptide pool as a positive control. The Negative (Unstimulated) Control is an essential component of an ELISpot assay as it helps determine the non-specific signal or background caused by cytokines necessary for the growth and proliferation of PBMCs. To accurately account for this non-specific effect, a subtraction method is employed. To quantify the specific immune response, the mean Spot Forming Units (SFUs) generated by the Negative control wells are subtracted from the SFUs generated by all the wells on the plate. This subtraction allows for the distinction between the specific immune response induced by the antigen of interest and the background signal resulting from cytokines present in the unstimulated control wells. A Mann-Whitney Test was performed to compare the mean no. of SFUs/10^6^ cells developed for each experimental peptide with that of the CMV positive control. The peptide ENDIKPKF (p=0.0417) was identified as the only neoantigenic peptide candidate that satisfied the set parameters for a positive response including p<0.05. This peptide (ENDIKPKF) exhibits a response which is approximately 2 folds greater than the response shown by the CMV positive control and 5 folds greater than the response shown by the unrelated peptide stimulated cells (**Figure 5**).

## 4 Discussion

Chimeric RNAs generated through chromosomal rearrangements (translocations, deletions, duplications and inversions), trans-splicing or read-through transcription have been proposed as reagents for developing tumor vaccines (16). Neoantigens generated from fusion transcripts have been reported to be better candidates for developing tumor vaccines because they are usually associated with significantly higher immunogenic potential than point mutation, SNV or in-del based neoantigens (3). Unique junctions formed in the chimeric RNAs that are translated can generate tumor-specific neoantigens, which can be exploited to design tumor vaccines for peptide-mediated T-cell activation and immunotherapies targeting cancer cells (3, 16). Our data suggests that chimeric RNAs are prevalent in breast tumors, provide a large number of novel fusions and generate immunogenic peptides that can elicit CD8+T cell responses, thus providing an expanded repertoire for development of breast cancer vaccines.

Breast cancer has low mutational burden, and therefore provides limited opportunities for peptide vaccine development. The chimeric RNAs that we uncovered, and the relatively large number of associated immunogenic peptides, open the door for cancer vaccines in these tumors with relatively fewer somatic mutations. The majority of the fusions discovered in our set of 75 cancer cases showed low frequency (present in 1-2 patients, ≤ 3% of the MDACC cohort). This is consistent with data from the TCGA Pan Cancer dataset that similarly noted that the overwhelming majority of fusions were private (17). Using computational approaches, the TCGA Pan Cancer study also determined the relative immunogenicity of neoantigens generated from fusions and reported that neopeptides derived from private fusions appeared to be more immunogenic than candidate neoantigens derived from highly frequent fusion events. While intriguing, these data lack direct *in vitro*/*in vivo* validation and thus the relationship between the frequency with which neoantigens are identified in the population and the ability to elicit a robust immune response remains unclear. Our data shows that some chimeric RNAs, such as *NSFP1*-LRRC37A2, occur at frequency in line with other therapeutic targets such as HER2/neu in breast cancer and EGFR in lung cancer, opening the door to an “off the shelf” peptide vaccine targeting tumors with these alterations, similar to targeted therapeutic strategies in breast and lung cancer.

In order to increase sensitivity and specificity of fusion discovery, we employed a unique strategy that incorporated two filters to significantly decrease the false positive and false negative rates of fusion detection. Focusing exclusively on the split reads crossing fusion junctions that are associated with discordant paired end reads bringing together two independent genes to extract chimeric RNAs that are not present in normal breast tissue we reduced the false positive rate. Including fusions that are present in adjacent normal samples (typically excluded by other ‘fusion callers’) and absent in normal breast tissue from cancer free patients, we significantly decreased the false negative rates of fusion detection. Additionally, this approach excludes chimeric RNAs that may be found in normal cells that have no impact on tumorigenesis or cancer progression (18). A number of fusion callers have been developed and published to extract fusion junctions from chimeric RNAs from RNAseq. Brian et al. and Trung et al. have each compared and benchmarked 15 gene fusion identification tools which are contingent on the accuracy of the transcriptome mapping (19, 20). Read length, quality scores and number of reads supporting each fusion were reported as the top limitations associated with fusion callers using short reads (21). *De-novo* assembly-based approaches yielding longer contigs have been reported to reduce limitations of short-read alignment but are computationally intensive (20–22). SeekFusion, developed by Balan et al. is designed to leverage *de-novo* assembly and alignment based approaches to increase the accuracy utilizing PCR-UMI-based amplicon RNA-Seq (23). Taking in to account the extensive body of prior work on fusion callers we used a multi-layered strategy to minimize false positives and false negatives. The key elements used include 1) de-novo assembly of RNA-seq data using the CLC Genomics Workbench 20 (Qiagen) to reduced false positives from shared repeat sequences on the genome; 2) utilized filters for removal of false positives from mis-mapping of reads to shared sequences in gene family members and/or pseudogenes when they exist (3, 24); and 3) relied heavily on fusions supported by split reads in multiple samples reported through other fusion callers from independent datasets (i.e. TCGA).

With an ultimate goal of identifying immunogenic peptides antigens that are broadly shared in breast cancer patients, we selected the *NSFP1-LRRC37A2* fusion transcript based on its frequency in tumor samples (found in 12% of samples tested) and 5 samples in the TCGA breast cancer dataset. *LRRC37A2* and *NSFP1* were previously predicted by the ChimeRScope pipeline to generate a fusion transcript in the opposite orientation (*LRRC37A2*-*NSFP1*) in a natural killer cell line (25). However, the data did not report the fusion junction site or exon boundaries due to poor sequence quality of the amplified PCR product (25). Increased read-depths made possible by decreased costs for RNA-seq applications have uncovered an increasing number of non-genetic gene fusions arising from intergenic cis- or trans-splicing that are emerging as new biomarkers and therapeutic targets for cancer (26). The *NSFP1-LRRC37A2* fusion is consistent with a transcriptional read through of the *NSFP1*-pseudo gene truncated at Exon 13 into *LRRC37A2* located immediately 3’ followed by a Cis-splicing event between *NSFP1* [Exon 13] and Exon 2 of *LRRC37A2* (**Figure 1**; **Supplemental Figure 1**). The relatively high degree of recurrence (12% in 75 patients) in 3 subtypes of breast cancer in our study and 5 subjects in TCGA breast tumor cohort makes it a highly attractive candidate for targeted therapies. The relatively low read numbers supporting the *LRRC37A2*-*NSFP1* fusion junction (average of 217-274 reads across the 75 samples) validated through PCR suggests that the fusion is likely present in a small subpopulation of cells in the tumor samples. Cai et al. and Carter et al. (27, 28) using clonal mutation analysis also report that tumor purity, heterogeneity and ploidy can result in variable cancer cell fractions in samples from cancer patients. However, if the fusion resulted from non-genetic fusions such as the one reported here they will not have corresponding DNA changes that are needed to compute CCF (cancer cell fraction) for each mutation.

Gene fusions have been reported to function as tumorigenic events in 16.5% of cancers and appear to be druggable in 6% of cases. The recurrent fusions commonly found associated with breast cancer and the potential impact of these in the development of new therapies for cancer is discussed by Loo et al. Gao et al. (29, 30). The most significant recurrent fusions reported from breast malignancies that could be benefit from targeted therapies as therapeutic vulnerabilities include *ESR1-CCDC170*, ESR1 exon 6 fusions, *BCL2L14-ETV6*, *ETV6-NTRK3* and *MYB-NFIB*. *ESR1-CCDC170* and ESR1 exon 6, have been reported to result in estrogen resistance and metastatic transformation in Luminal B breast cancer (31–33). *BCL2L14-ETV6* found in 6-12% of TNBC (34). *BCL2L14-ETV6* fusions reported in TNBC has been shown to result in EMT and paclitaxel resistance (35). 83% of a rare type of TNBS (adenoid cystic carcinomas (ACC) of the breast) carry the *MYB-NFIB* fusion (36). *ETV6-NTRK4* has been reported in secretory breast carcinoma (SBC). ETV6-NTRK3 and MYB-NFIB have been established to be cancer drivers (37, 38). Kinase fusions are currently being evaluated in breast cancer clinical trials and on-going mechanistic investigation is exposing therapeutic vulnerabilities in patients with fusion positive disease.

The *NSFP1*-[Exon-1-13]-KFPRKLYFLH C-terminal truncation and MISNQ-*LRRC37A2*-[Exon-2-14] N-terminal truncation together was found to generate 15 predicted immunogenic neoantigens with the potential to be processed and presented by 28 different MHC Class I alleles with a binding affinity of IC50<500nM. Out of the 15 peptides predicted to be immunogenic from the fusion junction, 8 peptides showed binding affinity (IC50<500nM) to the tested HLA Class of HLA-C*07:02. The peptide ENDIKPKF which showed the highest binding affinity (IC50 = 39) among all the peptides predicted to bind to HLA-C*07:02 was the only candidate which, satisfied the p<0.05 cutoff in the ELISpot assay (39).

In summary, we describe an untapped framework for discovery of neoantigens in breast cancer, generated through novel ORFs created from intergenically spliced mRNA transcripts. This novel pool of neopeptides broadens the opportunities for development of vaccines in breast cancer.

## Data availability statement

The original contributions presented in the study are publicly available. This data can be found here: NCBI, accession number PRJNA1004862.

## Ethics statement

The studies involving humans were approved by Institutional Review Board at MD Anderson Cancer Center under the protocol PA-16-0112. The studies were conducted in accordance with the local legislation and institutional requirements. Written informed consent for participation in this study was provided by the participants’ legal guardians/next of kin.

## Author contributions

BM, IB and PG conceptualized and designed the study. BM, SR, MC, AB, MR and HR contributed to the data acquisition and interpretation as well as in methodology and analysis. CA selected patient samples and oversaw the assembly of patient sample cores that were used for RNA extraction. BM, SR, MC, MR, AB, RK, IB and PG were major contributors in writing, review and editing the manuscript. All authors listed have made a substantial, direct, and intellectual contribution to the work and approved it for publication.
