# Supplementary material for: Chimeric RNAs reveal putative neoantigen peptides for developing tumor vaccines for breast cancer
Source: Front Immunol. 2023 Sep 6;14:1188831. doi: 10.3389/fimmu.2023.1188831 (PMC10512078; doi:10.3389/fimmu.2023.1188831)
Supplement: Supplementary file 1 [file DataSheet_1.docx]

Supplementary Material

Chimeric RNAs Reveal Putative Neoantigen Peptides for Developing Tumor Vaccines for Breast Cancer

## Supplementary Figures

**Supplementary Figure 1: Predicted *NSFP1* [Exon 1-13]-*LRRC37A2* [Exon 2-14] fusion transcript cDNA sequence.**

The consensus sequence for the junction-crossing reads was analyzed through BLAT using the UCSC Genome Browser to extract the fusion cDNA sequence shown above. The sequence contributed by *NSFP1* is shown in (blue) and *LLRC37A2* is represented in (red). The exon 13 junction on *NSFP1* and exon 2 junction of *LRRC37A2* are underlined.

**
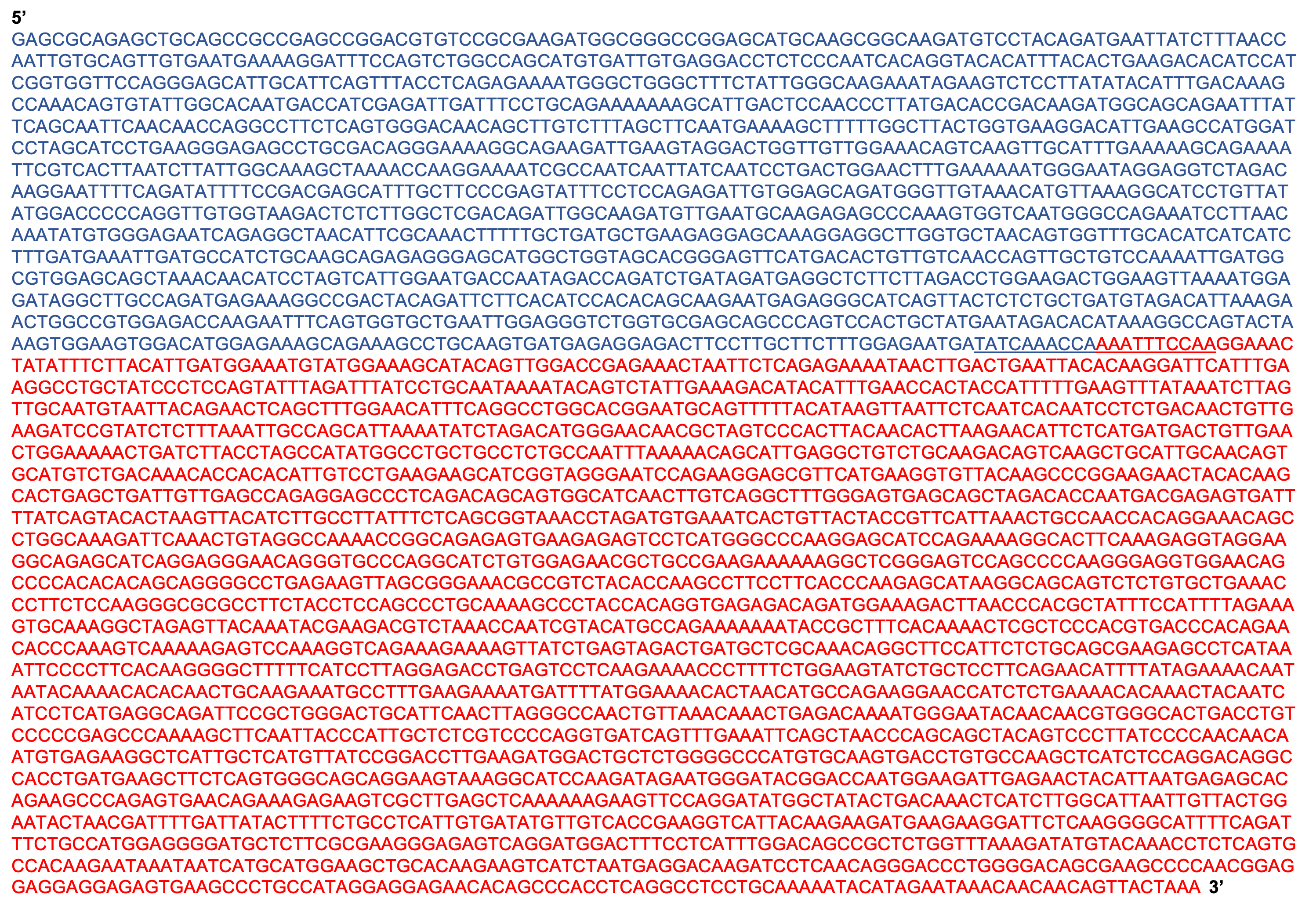
**

**Supplementary Figure 2: Schematic of the stimulation of PBMCs with predicted immunogenic peptides and IFN-y ELISPOT validation.**


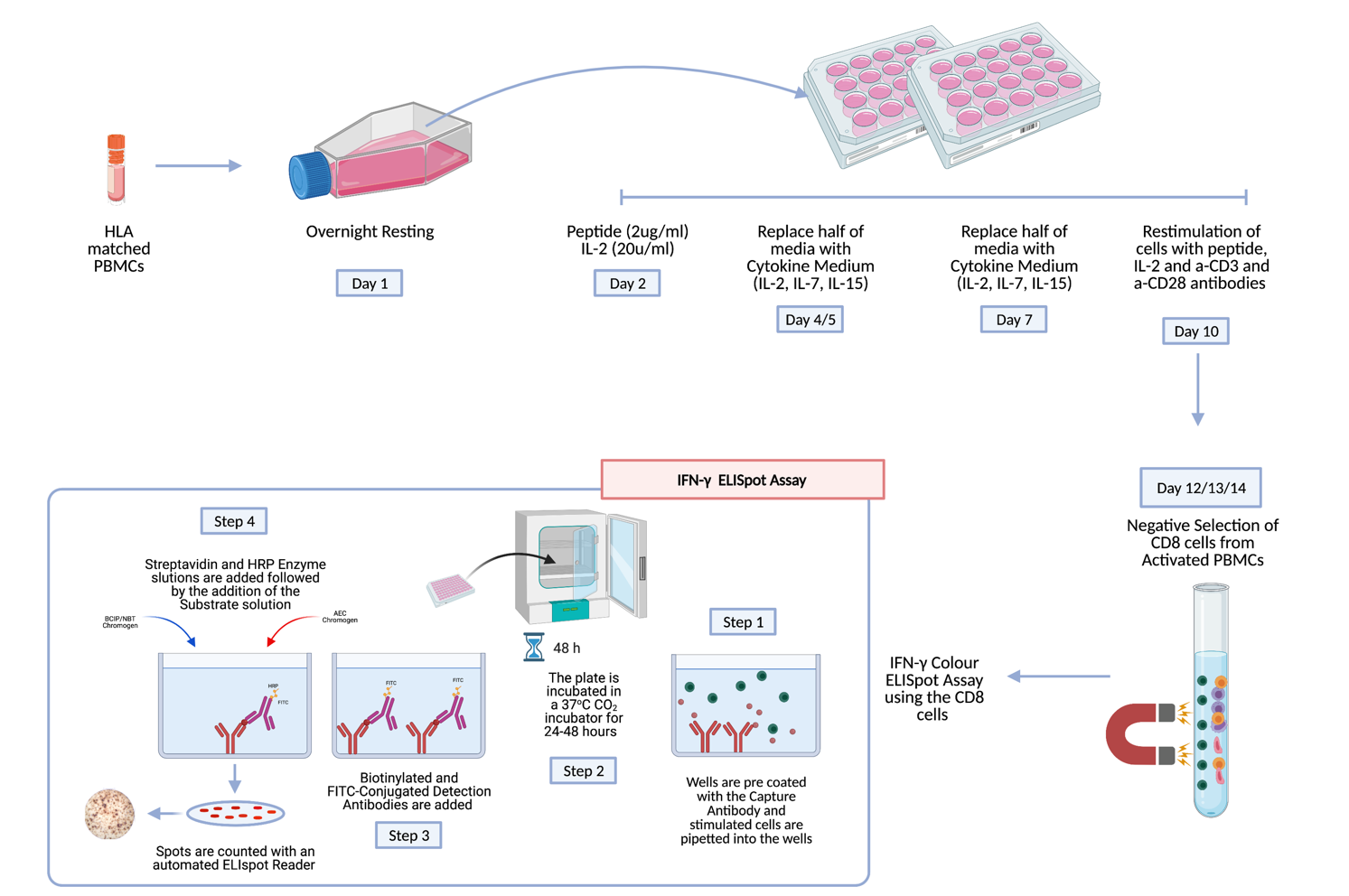


## Supplementary Tables

**Supplementary Table 1:** **Unique HLA binders of the immunogenic neoantigens from *NSFP1* and**  ***LRRC37A2* Truncations.**

Wild-type amino acids are colored (black), amino acid residues from the NSFP1-Truncation are colored (red) and residues from the LRRC37A2-truncation are colored (blue) respectively.

| **#Unique HLA-Binders *NSFP1***: [**FLASLENDIKPKFPRKLYFLH**] | |
| --- | --- |
| HLA-B*42:01 | HLA-C*03:03 |
| HLA-B*08:01 | HLA-A*24:03 |
| HLA-A*32:07 | HLA-B*15:03 |
| HLA-A*68:23 | HLA-A*23:01 |
| HLA-B*44:01 | HLA-A*24:01 |
| HLA-B*07:02 | HLA-B*27:02 |
| HLA-C*14:02 | HLA-C*07:02 |
| HLA-B*53:01 | HLA-A*30:01 |
| HLA-C*08:02 | HLA-A*68:23 |
| HLA-B*07:01 | HLA-A*32:07 |
| HLA-B*15:02 | HLA-A*33:01 |
| HLA-C*07:02 | HLA-A*68:01 |
| HLA-A*32:15 | HLA-B*44:01 |
| HLA-C*03:04 | HLA-C*12:03 |
| **#Unique HLA-Binders: *LRRC37A2*** [**MISNQNFQGNYISYID**] | |
| HLA-A*30:02 | HLA-B*15:01 |
| HLA-A*32:07 | HLA-C*03:03 |
| HLA-A*32:15 | HLA-C*12:03 |
| HLA-A*68:23 |  |
